# Barriers and Potential Improvements for Needle and Syringe Exchange Programs (NSPs) in China: A Qualitative Study from Perspectives of Both Health and Public Security Sectors

**DOI:** 10.1371/journal.pone.0130654

**Published:** 2015-06-26

**Authors:** Fung Kuen Koo, Xi Chen, Eric P. F. Chow, Jun Jing, Jun Zheng, Junshi Zhao, Lei Zhang

**Affiliations:** 1 Sydney Nursing School, The University of Sydney, Sydney, NSW, Australia; 2 Hunan Provincial Centers for Disease Control and Prevention, Hunan, China; 3 The Kirby Institute, UNSW Australia, Sydney, NSW, Australia; 4 Central Clinical School, Faculty of Medicine, Nursing and Health Sciences, Monash University, Melbourne, VIC, Australia; 5 Melbourne Sexual Health Centre, Alfred Health, Melbourne, VIC, Australia; 6 Research Center for Public Health, Tsinghua University, Beijing, China; University of Nebraska Medical Center, UNITED STATES

## Abstract

This study explores the acceptability, the barriers to the implementation of needle and syringe exchange programs (NSPs) and the potential improvement strategies in China from the perspectives of governmental health and public security officials. Purposive sampling was used for recruitment of participants who had been involved in NSPs implementation. Semi-Structured individual interviews were conducted in Mandarin to address three aspects of NSPs: (1) participants’ attitudes towards NSPs, (2) participants’ opinions on the effectiveness and barriers of NSPs, and (3) suggestions for improving the program. Content analysis was used to analyse the translated interview data. A total of 68 participants from 12 Hunan counties were interviewed (34 from each of the Bureau of Health and the Narcotic Division). Both groups recognised the importance and effectiveness of NSPs in HIV prevention, but public security officials regarded NSPs as a temporary intervention in place of punitive measures. Most health officials (32/34) regarded the main barriers to its implementation as administrative and structural, whereas participants from Narcotics Division (n=24) questioned the legitimacy of NSPs and concerned about the poor management of drug users’ risk behaviours. Close cooperation between the health and public security sectors, engagement of the drug user community and an enabling policy environment were reportedly to be critical for potential improvements of NSPs in China. Misconceptions about NSPs encourage drug users’ addictive behaviour, and an unclear leadership and insufficient support de-motivate the participants from the Bureau of Health and the Narcotics Division to actively support the program implementation.

## Introduction

Sharing of injecting equipment among injecting drug users (IDU) is a major mode of HIV transmission globally, especially in Southeast Asia, the Middle East, and Eastern Europe [1, 2]. Injection sharing has dominated HIV transmission in China in the past two decades [3]. According to the latest report from the National Narcotics Control Commission, the number of registered drug addicts increases gradually by year in China. As of 2010, the numbers of registered drug users reached 2.35 million, of which 56.6% was IDU [4]. HIV prevalence among IDU was 9.1% in 2010 [5] and about 38.5% of people living with HIV were injectors in China [4, 6].

Harm reduction interventions, particularly the needles/syringes programs (NSPs) and opioid substitution treatment (OST), have shown to be highly effective in reducing drug-related HIV transmission [2, 7]. In combination with antiretroviral therapy (ART), these programs can significantly reduce HIV incidence [8, 9]. However, implementation of harm reduction programs remain controversial in many parts of the world [10]. The Chinese government has declared ‘People’s war on Drugs’ in 2005 [11], aiming to strengthen narcotic control although forced detoxification and incarceration of drug users and re-education through labour camps. Official support for harm reduction came through a paradigm shift in the thinking of the Chinese government since 2006, in which, narcotics control requires a greater involvement from the public health sector and punitive measures, such as forced detoxification, are discouraged [12]. A change in governmental attitudes occurred from stigmatisation of drug users to the adoption of needle/syringe provision and methadone maintenance, as well as emphasis on improvements in HIV knowledge and consistent condom use for drug users [10, 13]. Providing clean needles and syringes to drug users through do not increase injection frequency [14–16], drug-related high risk behaviour [15, 17] and level of crimes [17, 18] among drug users. Consistently, NSPs in China has been shown to be highly effective in reducing HIV transmission, drug-related harm and at the same time being extremely cost-effective [19, 20]. Despite this, the development of NSP falls far behind that of OST (mainly methadone maintenance therapy [MMT]) in China [21]. MMT, which is misperceived by many as a curative therapy for addiction, often receives greater public support than NSP, which is also misperceived to encourage drug use.

A successful rollout of harm reduction programs often requires close collaboration between multiple government bodies, especially the health and public security sectors [22]. Despite a previous study argued that a lack of consensus among public security leaders and suggested increasing communication between police, government and public health officials in China [23], limited research explores the acceptability of the program implementation and the factors to facilitate cooperation between governmental health and public security officials. Through a qualitative approach, this study aims to explore the acceptability, the barriers in implementation and the possible improvements of the Chinese NSPs from both health and public security’s perspectives.

## Methods

**Fig 1:**
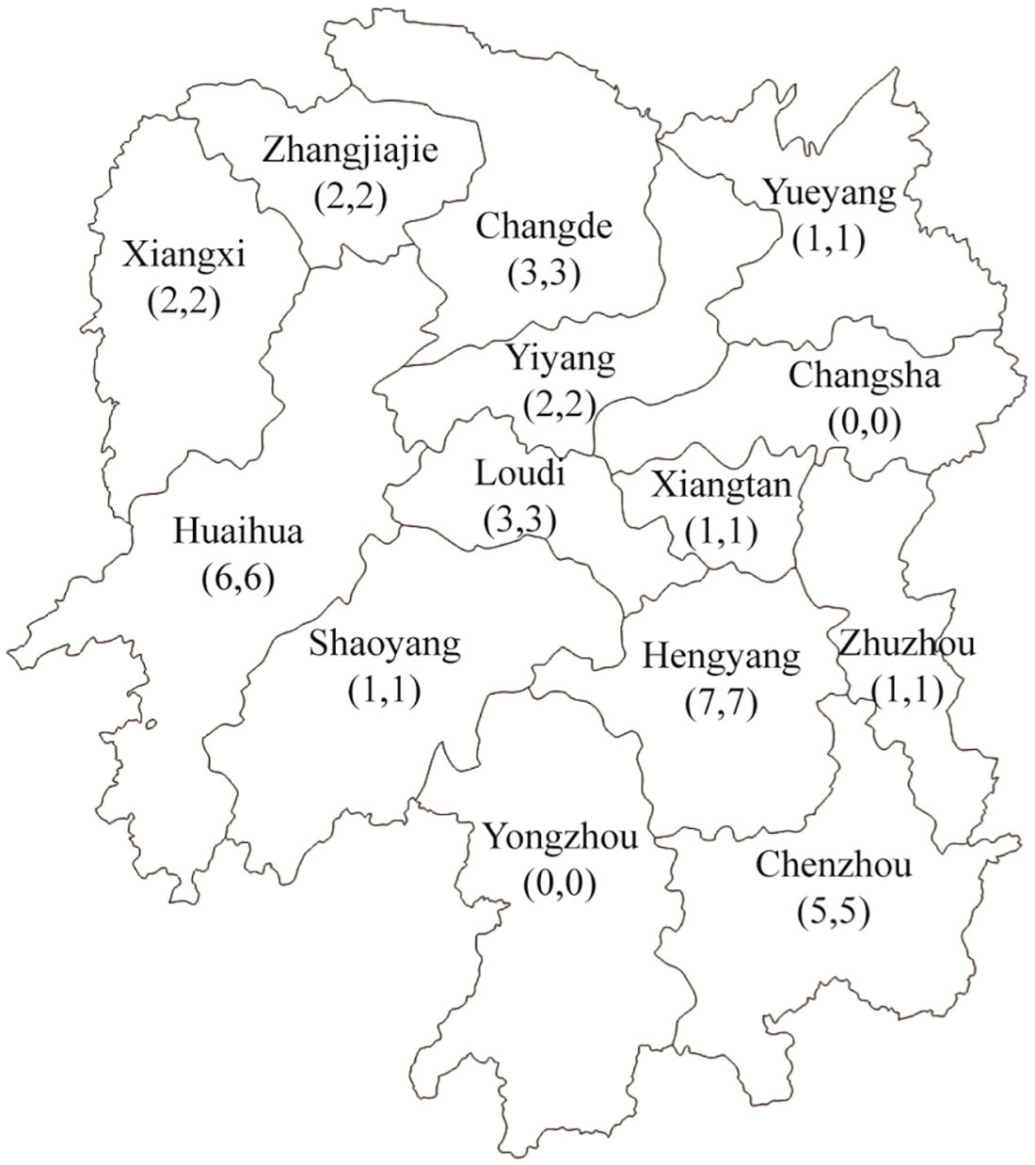
The distribution of study participants from ministries and health and public security in 12 counties of Hunan Province.

The study was conducted in 2008 in Hunan province, China. Hunan is located in South China, bordered by Guangdong and Guangxi provinces to the south and Guizhou province to the west. All three adjacent provinces are traditional drug-trafficking provinces with exceptionally high HIV prevalence levels among IDUs [24]. Hunan’s location has led to its role of channeling illicit drugs to other Chinese provinces [25]. Since 2003, the Hunan provincial Center for Disease Control and Prevention (CDC), Bureau of Health and Narcotics Division of Public Security (Narcotic Division) jointly implemented NSPs in 12 counties in Hunan Province. In order to explore the acceptability, the barriers and the attitudes from these two parties towards the current NSPs, purposive sampling was employed for this study to recruit participants who had been involved in its implementation. Strict criteria have been utilised in the recruitment process to facilitate diversity of feedback on NSPs. Sample recruitment was conducted from 34 participating NSP sites in the 12 Hunan counties (Fig 1). Potential participants who were full time staff and currently or previously working in the Bureau of Health and the Narcotic Division in Hunan were recruited for the interviews. Face-to-face individual interviews were conducted in Mandarin by well-trained interviewers from Hunan provincial CDC. Semi-structured interview questions were designed to address three aspects of NSPs: (1) participants’ attitudes towards NSPs; (2) participants’ opinions on the effectiveness and barriers of NSPs; and (3) suggestions for improving the program.

This study was approved by the Institutional Review Board at the Tsinghua University (Project Code: 0020120508). All interviews were conducted during the period between November 2009 and November 2010. Written informed consent was given to each participant prior to the interviews. Pseudonyms were used in the written report to ensure all participants’ confidentiality and anonymity. Interviews lasted between 60 and 90 minutes. Responses from the interviewees were audio-taped and collected by regular note taking. Interviews were continued until no further new information arose [26]. Tape-recorded interviews were transcribed into Chinese and then translated into English by the researchers. To ensure accuracy and to minimize the bias about representation of speech, five translated transcripts from participants were randomly audited and translated back to Chinese from English by two bilingual researchers (FKK, EPFC) who are fluent in both English and Mandarin, and an experienced Chinese-English translator. Content analysis was used to analyse the interview data. Three main phases of content analysis were involved: preparation, organisation (categorisation matrix development) and reporting of results. Two researchers (FKK, LZ) reviewed all data for content and coded for similarities and differences or exemplification of the identified categories [27] (S1 Table). Discussion among the research team members were conducted regularly at each phase of analysis for scrutiny and quality control (Table 1).

**Table 1:** Example of qualitative content analysis.

Step	Analysis process	Example quote
1	Meaning unit	There are a lot of potential issues. A lot of concerns. For instance, due to inadequate promotion, there is a widespread of fear among drug users towards NSPs. Moreover, think about the location, the locations of the NSP depots do not provide sufficient privacy, not enough. (a9)
2	Condensation	Due to inadequate promotion, there is a widespread of fear among drug users towards NSPs. Moreover, the locations of the NSP depots do not provide sufficient privacy. (a9)
3	Code	Difficulties in reaching the target drug users.
4	Category	Administrative and structural problems of the programs.
5	Theme	Barriers to NSP implementation.

Excerpts from an interview with a participant from the Bureau of Health (a9).

## Results

A total of 68 participants from 12 counties in Hunan were recruited and interviewed. This included 34 health officials from the Bureau of Health (code with ‘a’) and an equivalent number from the Narcotics Division (code with ‘b’) (Fig 1). The sex ratios of male-to-female were 2:1 and 3:1 respectively. The mean age for both groups was 40 years (Table 2). Further description about the characteristics of the participants was not revealed in this paper to prevent their identities from being connected with information.

**Table 2:** Distribution of age, sex and other general characteristic between the participants from the Bureau of Health and the Narcotics Division.

Demographic characteristics	The Bureau of Health (N = 34)	The Narcotics Division (N = 34)
Gender:		
Male	22 (65%)	26 (76%)
Female	12 (35%)	8 (24%)
Age (mean±SD)	40.3±3.19	39.6±3.46
Ethnics:		
Han	31 (91%)	32 (94%)
Non-Han	3 (9%)	2 (6%)
Education:		
College and above	20 (59%)	18 (53%)
Senior high and below	14 (41%)	16 (47%)

### Understanding of and attitudes towards NSPs

The vast majority of participants acknowledged the roll-out of NSPs in their jurisdictions, apart from one participant from the Narcotics Division replied, “no idea” (b2). Majority of the participants from both groups recognised the importance of NSPs provided by the Hunan provincial CDC. Findings revealed both groups believed NSP was important for HIV/AIDS prevention and control. Participants from Bureau of Health clearly demonstrated their understanding of the integrative services provided by NSPs, and those from the Narcotics Division emphasised the importance of NSP to reduce HIV transmission and enhance the self-awareness of HIV prevention among drug users. Notably, the group of Narcotics Division had a contradictory opinion on the effectiveness of NSP. The majority of the participants from the Bureau of Health (n = 29/34) agreed that NSP was an essential and integrated component of preventing and controlling HIV/AIDS, which “naturally includes needle and syringe exchange service, condom usage promotion, HIV/AIDS counselling and testing, health education and promotion and provision of free methadone [maintenance treatment]… (a29)”

Consistently, most of participants (n = 26/34) from the Narcotics Division also believed that the program such as NSPs were important because “HIV epidemic is far more fearsome and detrimental than a drug users (b11). They generally well endorsed the benefits of NSPs such as “reducing HIV transmission”, “being beneficial improvement to the [HIV] education” and “greatly enhancing the self-awareness of disease [HIV] prevention among drug users (b13)”. One of the participants even emphasised, “at the current stage it is very difficult for drug users to completely abstain from their drug addiction. The implementation of NSPs could be considered as a protective measure [HIV/AIDS] for drug users. (b31)”.

Whilst being questioned about the effectiveness of NSPs, 32 responses were obtained from each group. Twenty eight participants from the Bureau of Health believed that the program has been effective, whilst four participants stated otherwise. On contrast, although 19 participants from the group of the Narcotics Division recognised the effectiveness of NSPs, 13 others regarded it as either ineffective (n = 6/13) or having matching ‘pros and cons’ (n = 7/13). Furthermore, seven (n = 7/32) participants from this group did not support continuing NSPs in their local jurisdictions, while 22 (n = 22/32) participants supported its further development with the belief that “NSPs may reduce the burden of HIV and the numbers of drug users”. Three (3/32) did not express their views. A supportive public security officer stated: “when drug users can obtain clean and free [injecting] equipment, it prevents them from sharing [unclean] needles (b13)”. However, another participant, although supporting NSPs, shared a different view: “we actually do not fully accept NSPs, but the government and leaders give a green light to it, so we have to comply with their instructions within our work duties. In the long run, a strong crackdown on drug use must be considered as NSPs is only a temporary measure (b3)”. Among the public security officers who disagreed with the further development of NSPs, some (n = 3/7) believed that “for the Narcotics Division, it is not meaningful [to continue NSPs] as [its policy] it strictly combats illicit drug use, and thus showing no leniency to those who are caught (b25)”. Two others shared similar view and suggested to “replace NSPs with MMT (b1)”.

### Barriers to NSP implementation

Participants from both groups expressed different concerns about the implementation of NSPs. Thirty-two participants from the Bureau of Health indicated that administrative and structural problems of NSPs were the main barriers to its implementation. These included poor organisation coordination (n = 7/32), difficulty in managing drug users (n = 5/32), insufficient budget (n = 4/32), safety issues of staff at NSP sites (n = 3/32), unacceptance or misconception in the public (n = 4/32) and difficulties in reaching drug users (n = 3/32). Two health officials pointed out the urgency for resolving financial conflicts of interests between NSPs and other harm reduction programs such as MMT. One participant believed, “there is a limited budget for the [NSP] programs, staffs are therefore unwilling to participate due to the unattractive [financial] incentives (a34)”. Another participant added,

It is hard to manage drug users [as they do not follow the rules] and we do not have sufficient manpower and budget. More importantly, the administrative mechanism of the government and the relevant [administrating] departments require improvements [to handle the situation] (a6).

A substantial proportion of participants (n = 5/32) raised the concerns of safety and accessibility to NSPs. They explained, “due to inadequate promotion, there is a widespread of fear among drug users towards NSPs. Moreover, the locations of the NSP depots do not provide sufficient privacy (a9)”. Notably, three participants indicated that constant interference from the Narcotics Division of Public Security remains as a major barrier to access NSP sites because “the staffs from the Narcotics Division arrest the participating drug users [at NSP sites] (a17)”. Consistently, 14 of these health officials indicated that it was difficult and challenging to coordinate with the Narcotics Division.

Three participants also concerned about the safety of staffs since “the number of staff is limited; it is risky in dealing with drug users (a15)”. Another revealed: “drug users have poor conducts and often threaten the safety of staffs. It is difficult to manage them (a28)”. Participants also claimed that “the community will not accept [NSPs and the participating drug users]”. They believed “the drug users’ families also disagree with the service and engaging the peer workers in the services is lacking effectiveness (a17)”. In addition, participants suggested that “the mobility of drug users is high, it is a sensitive population and difficult to reach them… the budget [for the programs] is also insufficient (a10)”. A participant however believed that poor delivery of health education was a barrier as “there is no direct channel for the NSP staffs to effectively disseminate the health information to drug users, and drug users might not understand the risk of reusing and sharing needles with others (a31).”

Twenty-four participants from the Narcotics Division were concerned about the legitimacy of NSPs and poor management of drug users’ behaviours. They considered that NSPs is a program that encouraging drug users to continue injecting drug use and further create difficulties in dealing with them (n = 16/24). A substantial proportion from the Narcotics Division (n = 7/24) considered the idea of NSPs was contradicting with the current police’s job nature of encouraging drug users not to take drugs. Public misconception about NSPs (n = 1/24), differences in goals and duties of between the two Bureaus (n = 2/24), unwillingness of drug users to participate (n = 1/24) also posed significant barriers to the program. Some participants (n = 16/24) emphasised, “the drug users might think their drug using behaviour has been tacitly consented [by the government] (b5)” and in other words, “the program might weaken the willingness of drug users to stop taking drugs (b11)”. They also concerned about, “some drug users might use the NSP sites as a gathering point for drug consumption, drug trafficking and other illegal activities; and hence putting social order in jeopardy (b17)”. Furthermore, a participant claimed “to some extent, this program contradicts with the Narcotics Control Law in our country. It is in suspicion of indirectly encouraging or facilitating drug consumption (b17)”. Indeed, two participants pointed out the incompatibility between two Bureaus, “they have different work goals and mechanisms. The goal of the Narcotics Division involves stringent enforcement actions against drug use and trafficking but the Bureau of Health aims at disease prevention (b14)”. A participant cited on the opinions of the public, “it [NSP] hinders our daily operation and does not gain public understanding and identification (b18)”. Another participant also stated the barrier as “needles and syringes can be obtained at many places [chemist, private clinics], the number of people visiting the”Exchange Depots” is therefore scarce as drug users do not want to risk of being caught [by the police] (b21). Therefore, one participant concluded, “they [NSPs] cannot completely solve [the drug use problem] and keep the HIV epidemic under control (b28)”.

### Measures for program improvement

Concerning the improvement of NSPs, the group of Bureau of Health (n = 22) made three main recommendations: (i) involvement of other organisations or sectors to implement NSPs, (ii) a close cooperation between the Bureaus of Health and Narcotic Division; and (iii) engaging the drug user community and increasing the program budget. Regarding the involvement of other organisations or sectors (n = 16/22), some participants suggested to, “involve non-governmental organisations (NGOs) in the implementation of NSPs (a9)”. A participant explained,

NGOs have been conducting ongoing work for HIV prevention and have close contacts with high risk populations. It is more effective for NGOs to communicate with them and build mutual trust in their relationships. Moreover, some members from NGOs are HIV-infected drug users themselves, making it much easier for them to distribute needles and syringes than government employees. Also, NGOs provide a comparably less-tense environment and cause less stress for drug users (a31).

In addition, a participant further suggested, “while provincial CDC should be responsible [for operating the programs], the government should coordinate with Narcotics Division [to provide] more support and less interference (a30)”.

For those who supported the collaboration between two Bureaus (n = 5/22) mentioned, “We [Bureau of Health] need to get support from the Narcotics Division, local police, and social workers (a16).” A participant suggested,

Higher government administration should make effort in coordinating the Bureau of Health and the Narcotics Division, issuing legal documents to identify the responsibility of each department, unifying their goals and cooperating mechanisms. (a14)

Some participants (n = 3/22) recommended further involvement of the drug user community. One suggested to “make great efforts to equip peer educators [from the community], give them allowance, and widely distribute educational information related to NSPs; more advertisements in the community, more support and coordination from the Narcotic Division (a31)”. To achieve this, “increasing budgets for NSPs are inevitable”, as indicated by two other participants (a12 and a30). In addition, two health officials suggested that, “governmental level should enact related polices to promote social marketing of needles and syringes in community health clinics and chemists, and substantially increases the numbers of vending machines to enhance the coverage of NSPs (a13)”.

The Narcotics Division also suggested several ways to improve NSPs, including (i) policy development and increase program budget, (ii) enhancement of health promotion and education, and (iii) building a good collaboration with different governmental bodies. They also emphasised a feasible legal environment. Some participants (n = 8/18) repeatedly stated, for example, “National legislation should be established to foster a feasible legal environment for the cooperation between the public security and health sectors, to secure necessary operating budget for the NSPs (b28)”. A participant stated,

Drug users are required to register with the real names for needles and syringes exchange services. The name, gender and address of the participant are recorded. The program should aim at guiding the drug users to receive methadone as the primary treatment. The main emphasis of the work is to guide the drug users to quit drug … identify their own work responsibility, issue official documents to all middle and lower levels of government, departments of health and public security (b14).

Another important suggestion (n = 7/18) was the enhancement of health promotion and education, “It is essential to educate the drug users about the risks of needles sharing in order to reduce the HIV transmission (b24) and “more promotion in the community, so that the public understand the benefits of the program, and reduce discrimination and stigma (b26)”. “Developing a mutual goal” and achieving a “win-win situation” between Bureau of Health and the Narcotics Division was supported by participants (n = 4/18).

## Discussion

Although most study participant demonstrated positive attitudes towards NSPs, both groups expressed different views and attitudes on NSPs. The group of Bureau of Health were more focused on the administrative and structural issues of the program, and members of the Narcotics Division clearly expressed their concerns on the legitimacy of the NSP and the difficulties in its actual implementation. Responses from Narcotics Division expressed their reluctance to support NSP and only regarded the program as a transitional approach. They have strong view that MMT is far more effective than NSP and its rollout should eventually lead to the termination of NSPs in the near future. However, consistent with recent findings [28–30], fear of discrimination, lack of privacy, and police interference were identified from this study as the main hindrances of program participation.

In China, the successful implementation of NSPs requires a strong leadership and financial support from the central government. Our study indicated that the participants from the Narcotics Division were de-motivated to actively support the program implementation due to their misconception of NSPs would encourage drug users’ addictive behaviour. However, numerous evidences have shown the lack of causative relationship between receiving clean needle/syringes and increasing injection frequency [31, 32]. In contrast, comprehensive integration of HIV preventive measures, consisting of NSPs, substitution therapy, support and treatment for drug injectors, are crucial in curbing HIV epidemic [33]. Education and training for public security officers in relation to the benefits of NSPs would be essential for further development of NSP. Notably, under the current Chinese political hierarchy, the local governments are reluctant to assume the responsibility for NSP implementation, without the direct endorsement from their superiors. In an environment that misunderstanding about NSPs remains common in general population, local government officials, regardless of the Bureau of Health or Narcotics Division, will not encourage NSPs despite their knowledge of the effectiveness of program. The central Chinese government needs to assume a leading role in removing these misconceptions through mass health education and promotion campaigns. Fixed budgets should be allocated for NSPs as a part of the government-initiated national HIV strategies to resolve issues on under-staffing, insufficient revenue for commodities and lack of financial incentives for staff.

Echoing an earlier study conducted in Yunnan [23], mutual understanding and close collaboration between the two sectors of health and public security are essential for the implementation of NSPs. Our findings indicate that the lack of common goals and ineffective communication between the two government bodies may have substantially counteracted the impacts of NSPs. Consistent with previous studies [34, 35], both participant groups highlight the importance of collaborative partnership in policy development and program implementation. However, in contrast to the view of further NSP rollout held by public health officers, members from Narcotics Division insisted that the implementation of NSP operates against their job nature [10] and ongoing crackdowns on drugs will have a ‘chilling’ effect on the spread of drug use. This may create substantial barriers for drug users to access NSP. This significant finding has added extra insights and contrast to previous study [23]. The underlying reason for Narcotics Division’s opposition of NSP implementation is their regard of drug use as public security concern more than the pressure of meeting arrest quotas. Obviously, increasing communication between two government bodies is one of the main aspects for improvement [28]. Developing collaborative partnership by setting common vision and mission, establishing clear guidelines that defines roles and leadership, and reducing conflicts of interests are viable for the effective rollout of NSPs [36].

NSPs in China are a provider-initiated service and require active involvement from the drug users. Our findings revealed that distrust between NSP service provider (Bureau of Health) and clients (drug users) is an underlying barrier. This is particularly reflected by study participants’ concerns on poor management of drug addicts and the potential safety issues of NSP staff. Despite the development of a real name registration system for NSPs may be beneficial for systematic management of drug users for clinical diagnosis, healthcare linkage and provision, it could also be potentially used to provide ‘evidence’ on their illicit drug-use that leads to police confinement [37]. Re-establishing a mutual trust requires the development of appropriate policies and strategic plans that protect drug users’ privacy and confidentiality. On the other hand, strict regulations to forbid illegal activities on NSP sites, such as illegal trade of needles and syringes and exchange of illicit drugs, are essential to create a safe environment for the program staff [36]. Multi-sectoral training, policy analysis, regular program evaluation might be useful to harmonise laws and policies [32, 35] in order to foster a truly supportive environment in which NSP can achieve its best potentials on HIV prevention.

Strong community support by de-stigmatising drug users is essential for a long-term and sustainable implementation of NSPs. Adequate dissemination of accurate information on NSP is urgently needed to improve the general understanding and acceptance of the program. Consistent with previous studies [23, 28–31], stigma and discrimination toward drug users are also reported by both participating groups as their major concerns. The tension may be resolved by health education and promotion to drug users and the general public. Health education for drug users should not merely restrict to the availability and accessibility of clean needles and syringes, but also focus on enhancing individuals' motivation to their behavioural change, providing concrete strategies to risk reduction, and reinforcing positive behavioural change [38]. For the general public, media advocacy has been found to be an effective way in raising public awareness of and support for community drug-prevention programs [36, 39]. Creating a community environment that is friendly towards drug users plays an essential role in exerting long-term influences on drug users.

This qualitative study has several limitations. First, only basic but sufficient participants’ demographic backgrounds have been presented in this paper in order to ensure that participants remain unidentifiable with the provided demographic description. Second, participants were recruited from different counties in Hunan and therefore may not be representative of all governmental health and public security officials in China. Third, specific responses such as attitudes towards or misconception of NSP among the drug users who have utilised this service and the general public have not been explored specifically in this study. Fourth, typical Chinese communication style increased the difficulties in translation and interpretation of the responses [40–42]. Some participants preferred to answer questions in an indirect way, in which “meaning lies beyond words’ [41, 43]. Therefore, interviewers and investigators discussion have been conducted at each stage of transcription, translation and data analysis to provide a form of member checking.

## Conclusion

Misconceptions about NSPs encourage drug users’ addictive behaviour, an unclear leadership and insufficient support may de-motivate government officials from the Bureau of Health and the Narcotics Division to actively support the program implementation. Education and training, mutual trust in service providers and clients, close collaboration between the two sectors of health and public security are essential for the successful implementation of NSPs.

## Supporting Information

S1 Table Examples of the analysis of the contrast between positive and negative responses towards NSP among participants from bureau of health and narcotics division. (PDF)
