# Supplementary material for: Barriers and Potential Improvements for Needle and Syringe Exchange Programs (NSPs) in China: A Qualitative Study from Perspectives of Both Health and Public Security Sectors
Source: PLoS One. 2015 Jun 26;10(6):e0130654. doi: 10.1371/journal.pone.0130654 (PMC4482722; doi:10.1371/journal.pone.0130654)
Supplement: S1 Table — (PDF) [file pone.0130654.s001.pdf]

S1 Table. Exemplary analysis of the contrast between positive and negative responses towards NSP among participants from bureau of health and narcotics division.

**Positive attitudes**	**Negative attitudes**	**Analytical Comments**
Bureau of Health:<br>*(responses=28)*<br><br>Examples:<br>NSP was important for HIV/AIDS prevention and control …<br><br>NSP was an essential and integrated component of preventing and controlling HIV/AIDS …<br><br>The NSE program provides free blood test for HIV, syphilis and HCV [Hepatitis C Virus] …<br><br>It provides needle and syringe exchange service, health promotion and education …<br><br>NSP includes needle and syringe exchange service, promotion for using condoms, HIV/AIDs enquiry and blood test service, health education and promotion, supply of free Methadone, sexual transmitted disease referrals, antivirus treatment …<br><br>The good points of the programs were … naturally includes needle and syringe exchange service; condom usage promotion; HIV/AIDS counselling and testing; health education and promotion and provision of free methadone [maintenance treatment] …<br><br>The programs were quite effective …	***Bureau of Health:***<br>*(responses=4)*<br><br>Examples:<br>NSP is useful to a certain extent but the outcomes was not very ideal …<br><br>If you look at the cost and effect, the result was not very reasonable …<br><br>The program was not effective … it can be related to cultural beliefs and public misconception of the program from the public	Positive comments focussed on the program services and outcomes<br><br>Negative comments focussed on the external factors or pressure

The Narcotics Division: (*responses=19)*<br><br>NSP can reduce HIV transmission and enhance the self-awareness of HIV prevention among drug users.<br><br>There are many benefits to implement NSPs including: "reducing HIV transmission", "being beneficial improvement to the [HIV] education" and "greatly enhancing the self-awareness of disease [HIV] prevention among drug users.<br><br>NSPs were important because HIV epidemic is far more fearsome and detrimental than a drug users …<br><br>At the current stage it is very difficult for drug users to completely abstain from their drug addiction. The implementation of NSPs could be considered as a protective measure [HIV/AIDS] for drug users …<br><br>NSPs may reduce the burden of HIV and the numbers of drug users ...<br><br>When drug users can obtain clean and free [injecting] equipment, it prevents them from sharing [unclean] needles.	***The Narcotics Division :*** (*responses=13)*<br><br>NSP was ineffective … NSPs did not think about the 'pros and cons'…<br><br>We actually do not fully accept NSPs, but the government and leaders give a green light to it, so we have to comply with their instructions within our work duties …<br><br>Our role is to catch the drug addicts …<br><br>In the long run, a strong crackdown on drug use must be considered as NSPs is only a temporary measure …<br><br>For the Narcotics Division, it is not meaningful [to continue NSPs] as [its policy] it strictly combats illicit drug use, and thus showing no leniency to those who are caught …<br><br>I suggested that we need to replace NSPs with MMT …	Positive comments focussed on the program outcomes<br><br>Negative comments focussed on their own subjective beliefs about NSPs or own concerns, particularly the clashes with their job nature/work duties
